# Impact of Wind on Rainfall Measurements Obtained from the OTT Parsivel^2^ Disdrometer

**DOI:** 10.3390/s25206440

**Published:** 2025-10-18

**Authors:** Enrico Chinchella, Arianna Cauteruccio, Luca G. Lanza

**Affiliations:** 1 Department of Civil, Chemical and Environmental Engineering, University of Genova, 16145 Genoa, Italy; enrico.chinchella@edu.unige.it (E.C.); arianna.cauteruccio@edu.unige.it (A.C.); 2 WMO Measurement Lead Centre “B. Castelli” on Precipitation Intensity, 16145 Genoa, Italy

**Keywords:** adjustment, atmospheric precipitation, computational fluid dynamics, drop size distribution, Lagrangian particle tracking, measurement accuracy, numerical simulation, wind-induced bias

## Abstract

**Highlights:**

**What are the main findings?**

- Wind affects rainfall measurements taken by the OTT Parsivel^2^ disdrometer, with significant under- or overestimation depending on wind speed and direction.
- Numerical simulation enables the wind-induced bias of disdrometer measurements to be quantified in terms of site-independent catch ratios.

**What is the implication of the main finding?**

- Raw measurements should be adjusted using the derived catch ratios, based on the measured drop size distribution, wind speed and direction.
- Positioning of the instrument to align with the prevailing wind direction is crucial to achieve acceptable accuracy in rainfall measurement.

**Abstract:**

The impact of wind on precipitation measurements from the OTT Parsivel^2^ optical transmission disdrometer is quantified using computational fluid dynamics simulations. The numerical velocity field around the instrument body and above the instrument sensing area (the laser beam) shows significant disturbance that depends heavily on the wind direction. By computing the trajectories of raindrops approaching the instrument, the wind-induced bias is quantified for a wide range of environmental conditions. Adjustments are derived in terms of site-independent catch ratios, which can be used to correct measurements in post-processing. The impact on two integral rainfall variables, the rainfall intensity and radar reflectivity, is calculated in terms of collection and radar retrieval efficiency assuming a sample drop size distribution. For rainfall intensity measurements, the OTT Parsivel^2^ shows significant bias, even much higher than the wind-induced bias typical of catching-type rain gauges. Large underestimation is shown for wind parallel to the laser beam, while limited bias occurs for wind perpendicular to it. The intermediate case, with wind at 45°, presents non negligible overestimation. Proper alignment of the instrument with the laser beam perpendicular to the prevailing wind direction at the installation site and the use of windshields may significantly reduce the overall wind-induced bias.

## 1. Introduction

Non-Catching type Gauges (NCGs), including disdrometers, are increasingly used to measure the microphysical properties of rainfall. Optical transmission disdrometers detect the size and fall velocity of each individual raindrop in flight by measuring the obstruction to a generated light beam, therefore providing the drop size distribution (DSD) of the observed rain event. This non-contact measurement technique is also particularly promising for various applications where integral rainfall variables, such as rainfall intensity (RI), radar reflectivity (Z) and kinetic energy (KE), are obtained by processing the raw measurements.

In addition to instrumental bias, which can be quantified through accurate laboratory calibration [1], disdrometers are susceptible to environmental sources of measurement bias. These are due to environmental conditions at the measurement site and include, for example, the impact of sunlight, lighting, wind, and atmospheric pollution. The present work focuses on the impact of wind, recognised as the primary environmental factor inducing bias in rainfall measurements [2].

Indeed, the instrument body behaves like a bluff-body obstacle to the wind, generating strong velocity gradients and flow deformation near its sensing area (i.e., the laser beam in the case of optical transmission disdrometers). Such aerodynamic disturbance affects raindrop trajectories, which may be diverted from or towards the instrument’s sensing area depending on their size and fall velocity. This phenomenon is well documented in the literature for both catching gauges (CGs), which are traditionally used for measuring rainfall and snowfall, and NCGs, though less frequently quantified for disdrometers [3].

One example is the work of Capozzi et al. [4], in which the wind-induced bias in rainfall measurements taken by the Laser Precipitation Monitor (LPM) [5]— an optical transmission disdrometer manufactured by Thies Clima, Adolf Thies GmbH & Co. KG, Göttingen, Germany —was estimated in situ by comparing it with a collocated tipping-bucket rain gauge. Evidence of artefacts in the measured DSD was observed in data from a stationary Parsivel optical transmission disdrometer [6] (manufactured by OTT Hydromet GmbH, Kempten, Germany) when compared with data from an identical instrument with an automatic variable orientation and tilting mount that was used to continuously align it with the wind direction [7]. Similar discrepancies were noted in measurements from the Thies LPM, even at limited wind speed. Upton and Brawn [8] installed two such instruments with two different orientations (rotated by 90°) and reported differences of up to 20% in the total number of detected raindrops.

Wind-induced biases were also reported for the Two-Dimensional Video Disdrometer (2DVD)—manufactured by Joanneum Research, Graz, Austria — by Greenberg [9], who noted an underestimation of precipitation when part of the instrument’s sensing area received no precipitation under specific wind conditions. Further studies conducted by Testik and Pei [10] on an improved version of the 2DVD showed that the effect of wind on precipitation is still present, affecting not only the measured amount and intensity, but also the DSD.

Methods to quantify the wind-induced bias in rainfall measurements include field comparisons against reference shielded gauges (see, e.g., [11]), wind tunnel (WT) experiments (see, e.g., [12] and the references therein) and numerical simulations (see, e.g., [13]). The main obstacle to quantifying the wind-induced bias of NCGs is their complex, usually non-radially symmetric geometry. Their aerodynamic behaviour depends heavily on the wind direction, which significantly increases the time and effort needed to evaluate the bias using field campaigns and WT experiments.

Instead, numerical simulation allows many different configurations to be easily investigated in terms of wind speed and direction, instrument geometry, and precipitation type (see, for example [4]), at an acceptable cost in terms of computational resources. Nešpor et al. [14] were the first to use a numerical approach to investigate the wind-induced bias for NCGs, applying it to the study of the 2DVD. However, the outer shape of the instrument considered in that study is now obsolete and differs significantly from the current version.

In their recent work, Chinchella et al. [3,15] quantified the wind-induced bias numerically for two widely adopted NCGs: the WXT520 (manufactured by Vaisala, Helsinki, Finland) electroacoustic precipitation sensor [16] and the Thies LPM. Various combinations of wind speed and direction were studied for both instruments, showing that liquid precipitation could be overestimated by up to 400% (WXT520) or not measured at all (Thies LPM). However, variability in instrument geometry and the measuring principles adopted makes it impossible to use a common adjustment function.

In this study, we use numerical simulations to quantify the wind-induced bias of rainfall measurements for the widely used OTT Parsivel^2^ optical transmission disdrometer and develop adjustment curves for use in practical applications. Catch ratios, which are a site-independent measure of instrument performance in windy conditions, are obtained as a function of raindrop size, wind speed, and direction. After assuming a sample DSD function and the relationship between its parameters and rainfall intensity, the wind-induced bias is quantified for relevant integral variables, such as RI and Z, and adjustment curves are provided.

The organisation of the paper is as follows: Section 2 describes the simulation framework, numerical setup and assumptions, while Section 3 reports the results in terms of the aerodynamic behaviour of the instrument and the impact on the fall trajectories of raindrops of different sizes. Section 4 discusses and quantifies the resulting wind-induced bias by calculating the catch ratios and their dependence on raindrop and wind characteristics. The bias on integral variables derived from raw measurements is also quantified in Section 4 and conclusions are drawn in Section 5.

## 2. Methodology

A two-step procedure was employed to evaluate the wind-induced bias for the OTT Parsivel^2^ in case of rainfall measurements. First, the velocity field around the instrument was obtained from Computational Fluid Dynamics (CFD) simulations involving different wind speeds and directions. Second, the effect of the resulting aerodynamic disturbances on raindrops was evaluated using a Lagrangian Particle Tracking (LPT) model. The instrument’s performance in windy conditions was evaluated by analysing the computed raindrop trajectories and how they interacted with the instrument body and sensing area.

### 2.1. The OTT Parsivel^2^ Disdrometer

**Figure 1:**
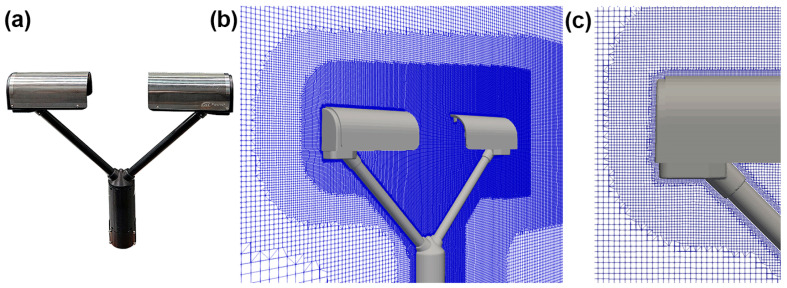
(**a**) The OTT Parsivel^2^ disdrometer, showing the emitter and receiver heads, as well as the main body, which holds the two supporting arms and contains the circuitry. (**b**) Cross-section of the mesh used to discretise the computational domain around the instrument body, taken along the Y = 0 plane. (**c**) Detail of the mesh refinement close to the instrument surface.

The Present Weather Sensor (PWS), manufactured by OTT HydroMet GmbH, Kempten, Germany and named Parsivel^2^ (an acronym for ‘Particle Size and Velocity’), is equipped with horizontally aligned laser emitter and receiver heads connected to the instrument’s main body by supporting arms (see Figure 1a). A laser diode is used to produce a thin light beam that is 30 mm wide and approximately 180 mm long, considering the part of the beam that is exposed to undisturbed (vertical) precipitation. The power of the laser beam is converted into an electrical signal by a photodiode in the receiver head. This signal is reduced by any hydrometeor or other opaque object crossing the beam and casting a shadow on the receiver. The light blockage—and therefore the signal reduction—is proportional to the size of the falling hydrometeor.

The fall velocity is obtained from the duration of the beam blockage (extinction). The combination of hydrometeor size and fall velocity, together with the measurement of local temperature, also makes it possible to identify the type of precipitation (e.g., drizzle, rain, snow, soft hail, hail and mixed) and to exclude objects other than hydrometeors that may cross the laser beam, such as insects, since the size-velocity relationship would not be consistent with that expected for hydrometeors (e.g., provided by Gunn and Kinzer [17]).

The instrument reports the diameter of raindrops in 32 classes, ranging from 0.2 to 8 mm (extended to 25 mm for solid hydrometeors). The fall velocity is also reported in 32 classes, spanning a range of 0.2 to 20 m s^−1^. The precipitation type is categorized in 8 classes, from drizzle to hail. The size and fall velocity distribution over the class binning, aggregated at 1 min resolution, is also used to calculate the intensity and amount of precipitation, visibility, the kinetic energy of precipitation and radar reflectivity.

The OTT Parsivel^2^ has a non-radially symmetric geometry that is significantly more complex than the cylindrical or semi-cylindrical shape of traditional catching-type rain gauges. Furthermore, the two heads containing the laser emitter and receiver are quite bulky and obstruct the wind significantly. This also implies that the wind-induced disturbance depends strongly on the wind direction.

The instrument’s operating manual acknowledges this fact and recommends aligning the laser beam perpendicular to the prevailing wind direction at the installation site. It also notes that glare and intense sunlight may impact the measurements. The same operating manual states that the accuracy of rainfall intensity measurements is ±5%, whereas this degrades to ±20% in the event of solid precipitation [6].

### 2.2. Fluid Dynamics Simulation Setup

The simulations were based on the numerical model of the instrument geometry, which was provided by the manufacturer. The Unsteady Reynolds Averaged Navier–Stokes (URANS) equations around the instrument body were solved numerically using the open-source OpenFOAM C++ library [18]. Simulations were run under the hypothesis of stationary turbulence characteristics until the steady state was reached. Assumptions about the physical properties of air are as follows: incompressible fluid, density of 1.2 kg m^−3^ and kinematic viscosity equal to 1.5 × 10^−5^ m^2^ s^−1^, with a free-stream turbulence intensity of 1%.

A structured mesh with variable refinement was used to discretise a simulation domain of 4 m (length) by 2.4 m (width) by 2 m (height) surrounding the instrument geometry, an example of which is shown in Figure 1b. The wind direction is along the longitudinal axis (X), with an upward pointing vertical axis (Z) and a transversal axis (Y) that is perpendicular to both X and Z.

Five different meshes were created by rotating the instrument inside the domain to simulate various wind directions from α = 0° to α = 90°, in increments of 22.5°. Here, α is the angle between the wind direction and the instrument’s main symmetry axis. In the configuration at α = 0° the X axis is parallel to the main symmetry axis of the instrument, therefore the wind first impacts one of the two heads, whereas in the configuration at α = 90°, the wind impacts the side of the instrument perpendicular to the laser beam. The origin of the reference system is in the centre of the instrument’s sensing area (the laser beam). The mesh has a variable cells size with a maximum of 0.05 m and a minimum of 0.75 mm near the instrument (see Figure 1c). This allows reproducing the fine details of the instrument body and correctly model the turbulence generated by flow interaction with solid surfaces.

Seven wind speed values (U_ref_) of 1, 2.5, 5, 7.5, 10, 15 and 20 m s^−1^ were simulated for each direction.

Preliminary simulations were run to assess the quality of the mesh resolution, using the calculated ratio (R_L_) of the integral length scale of turbulence to the grid length scale. For URANS simulations, an R_L_ value of at least 5 is required to ensure that the larger eddies, which account for 80% of the turbulence kinetic energy, are discretised by at least 5 cells [19]. We adopted the *k*-*ω* Shear Stress Transport (SST) model for turbulence, where *k* is the turbulent kinetic energy and *ω* the specific turbulent dissipation rate, therefore the integral length scale (L_0_) is obtained from Equation (1), while R_L_ is obtained from Equation (2).

(1) L0=k12Cμ·ω

(2) $$R_{L}=\frac{L_{0}}{\sqrt[{3}]{V_{c}}}\;$$

where the coefficient $C_{\mu }$ is equal to 0.09 and *V_c_* is the volume of the cell.

**Figure 2:**
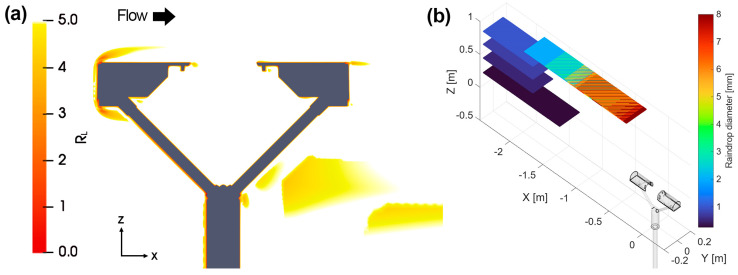
(**a**) Map of the R_L_ ratio at U_ref_ = 10 m s^−1^ for α = 0°, along the Y = 0 plane. Values of R_L_ are below the threshold only close to the instrument stagnation point and in its wake. (**b**) Visual representation of the particle seeding grids used (colour coded as a function of diameter in the image) for α = 0° and U_ref_ = 10 m s^−1^, above the instrument (also visible in the bottom right corner of the domain).

Figure 2a shows the map of R_L_ values obtained, indicating that the optimality criterion (R_L_ ≥ 5) is met across most of the domain, except in the vicinity of the instrument’s stagnation point (top left) and in the wake of the supporting arms.

The average value of the dimensionless wall distance (y+) obtained from preliminary simulations indicates, according to wind velocity, whether the mesh cells closest to the instrument surface are positioned in the buffer or the log-law layer. The near wall boundary conditions for k, ω and the turbulent viscosity (ν_t_) [20] were set at all solid surfaces using wall functions, independent of y+.

The non-orthogonality, skewness and aspect ratio (see, e.g., [21,22]) of the final meshes, containing between 6 and 8 million cells depending on wind direction, are listed in Table 1. The numerical simulation setup was validated in previous work for various geometries (see, e.g., [15]) using wind tunnel experiments. Due to the large meshes required the computational cost of these simulations was about 30,000 core hours.

### 2.3. Lagrangian Particle Tracking

Since the volume fraction of raindrops in the atmosphere is generally low, even at high rainfall rates [23], their presence does not affect the airflow around the instrument body. Furthermore, particle-to-particle interactions are very limited close to the ground, where the instruments are positioned. We therefore adopted an uncoupled LPT model, which is computationally inexpensive yet capable of modelling the interaction between the instrument, wind and precipitation.

In the uncoupled model, trajectories are obtained by solving the equation of motion (see, e.g., [12]), based on a stationary airflow velocity field (i.e., the steady-state solution), and by neglecting particle-to-particle interactions. Assuming a steady state condition for the wind field is also reasonable given typical field conditions. This is because the typical time interval required for a drop to cross the computational domain is between 0.1 and 1 s.

Raindrops are released into the computational domain from a regular grid with a width of 0.15 m and a length of at least 0.45 m, with a regular spacing of 2 mm. In undisturbed conditions, the drop released from the centre of the grid would reach the centre of the instrument laser beam. The grid length is increased for high wind speeds and small raindrop diameters. Depending on the specific combination of drop diameter, wind speed and direction between 60,000 and 400,000 particles were therefore released in the domain. Of these, only about 2.25% to 0.33% are expected to reach the instrument’s sensing area in undisturbed airflow conditions. This ensures that all trajectories that could potentially reach the instrument sensing area are included in the simulation. Figure 2b shows an example of the release grid for a wind speed of 10 m s^−1^ and various drop diameters.

A virtual surface, transparent to the wind but not to trajectories, was also included to model and visualise the instrument laser beam. Drops were then tracked until they impact on the instrument body, the instrument sensing area or exit from the domain boundaries. The simulations were then stopped once all released drops impacted one of the domain boundaries or travelled significantly below the instrument sensing area. In total 11 drop diameters were simulated, equal to 0.25, 0.5, 0.75 and from 1 mm up to 8 mm (with 1 mm increments).

An in-depth analysis on the use of a LPT model for simulating drop trajectories and an extensive wind tunnel validation is presented in the work of Cauteruccio et al. [12]. The drag coefficient formulation used in that work is implemented here for various ranges of the particle Reynolds number, as established a priori among those proposed in the literature by Folland [24] and formulated starting from data published by Beard [25] and Khvorostyanov and Curry [26]. Since an uncoupled approach was used, the computational cost for running the LPT model was about 500 core hours.

## 3. Simulation Results

CFD simulation results are shown in terms of maps of the normalized magnitude and vertical component of flow velocity (indicated with U_mag_/U_ref_ and U_z_/U_ref_, respectively) and the generated turbulent structures are visualised using the Q-criterion [27]. Fall trajectories are shown for sample raindrop sizes to visualize their deflection and the change in their vertical fall velocity.

### 3.1. CFD Simulation Results

**Figure 3:**
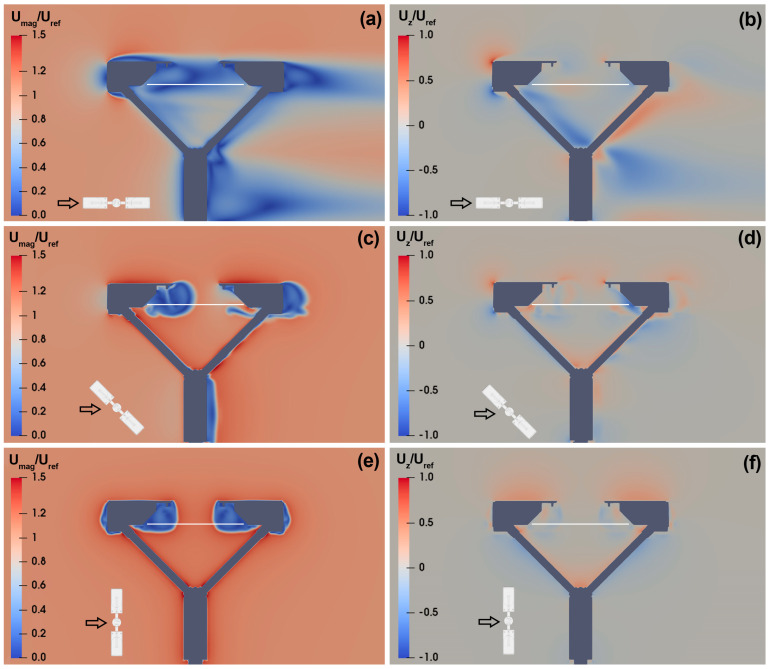
CFD simulation results for U_ref_ = 10 m s^−1^ and α = 0° (**a**,**b**), 45° (**c**,**d**), 90° (**e**,**f**). The maps show the normalized magnitude (**a**,**c**,**e**) and vertical component (**b**,**d**,**f**) of the airflow velocity along the Y = 0 plane, parallel to the laser beam. The white horizontal line shows the location of the instrument’s sensing area, while the small black arrow shows the wind direction relative to the instrument orientation.

This section synthesises the large numerical dataset obtained from CFD simulations by visualising sample cross-sections of the wind velocity field parallel to the instrument laser beam. This is done for an undisturbed velocity (U_ref_) of 10 m s^−1^ and wind directions α of 0°, 45° and 90°. The normalised magnitude of the flow velocity is shown in the left-hand panels of Figure 3 for each wind direction. The red and blue areas indicate higher and lower flow velocity than U_ref_, respectively. In the right-hand panels of Figure 3, the normalised vertical velocity is shown for each wind direction. Red zones indicate upward flow velocity components (U_z_/U_ref_ > 0), while blue zones indicate downward components (U_z_/U_ref_ < 0).

In Figure 3a,b, at α = 0°, one of the two heads is the first bluff-body obstacle to the flow. It generates accelerated zones that remains—for the most part—attached to the instrument body, generating a relatively thin boundary layer. The instrument’s sensing area (white horizontal line) is entirely contained in a low velocity zone, characterized by strong turbulence and recirculation (Figure 3a). In terms of vertical velocity components (Figure 3b), updraft is mostly concentrated upstream of the instrument body and in the wake of the downwind supporting arm. Meanwhile a significant downdraft zone is present in the wake of the first supporting arm. Near the instrument’s sensing area both updraft and downdraft occur due to turbulence and recirculation, although with limited intensity.

In Figure 3c,d, at α = 45°, the aerodynamic disturbance—in the form of a low velocity area—affects only a portion of the instrument’s sensing area, resulting in a strong velocity gradient at about half the length of the laser beam. The boundary layer produced by the instrument heads is not visible in this orientation except for its wake beyond the downwind head (Figure 3c). Vertical velocity components are less intense than in the previous case. However, their intensity close to the instrument’s sensing area are visibly higher, and more widespread than in the previous case, increasing the volume of the domain where the airflow deformation may affect raindrop trajectories (Figure 3d).

Finally, in Figure 3e,f, at α = 90°, the two heads show symmetric behaviour because they are equally impacted by the wind. However, in this case, the sensing area of the instrument that is usually exposed to precipitation is largely free from aerodynamic disturbance, while recirculation is mostly limited to the immediate vicinity of the two heads (see Figure 3e). Unlike the previous case, updraft occurs close to the two heads and near the instrument’s sensing area, albeit with a similar intensity (Figure 3f).

**Figure 4:**
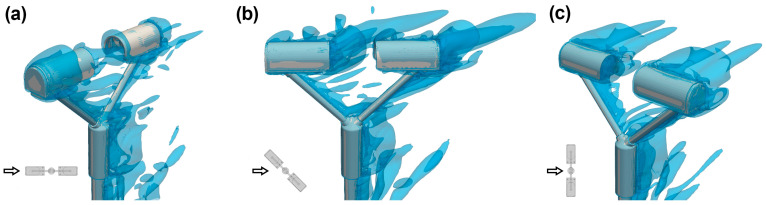
Visualization of the turbulent structures around the instrument body using the Q-criterion at U_ref_ = 10 m s^−1^ and α = 0° (**a**), 45° (**b**), and 90° (**c**). The small black arrow shows the wind direction relative to the instrument orientation.

Figure 4 visualises the turbulence structures developing near the instrument body using the Q-criterion. For α = 0° (Figure 4a), relatively small vortices are visible near the upwind head of the instrument that propagate towards the sensing area. At α = 45° and α = 90° (Figure 4b,c), larger vortices are generated. However, these vortexes are mostly advected away from the instrument’s sensing area, resulting in a lower overall impact.

Similar results were obtained at different wind velocities and directions. The recirculation and vertical velocity components near the sensing area decrease non-linearly from the 0° configuration to the 90° configuration, reaching a minimum at the latter.

### 3.2. Raindrop Fall Trajectories

Drops of different sizes are affected by wind to different extents depending on their Stokes number (Stk), which is defined as the ratio of a drop’s characteristic time to the characteristic time of the flow. Considerable deviation in trajectory is expected for small-size drops (Stk << 1), while large-size drops are almost unaffected (Stk >> 1). However, small-size drops are the most abundant in natural rainfall events and account for a substantial portion of the total precipitation volume.

**Figure 5:**
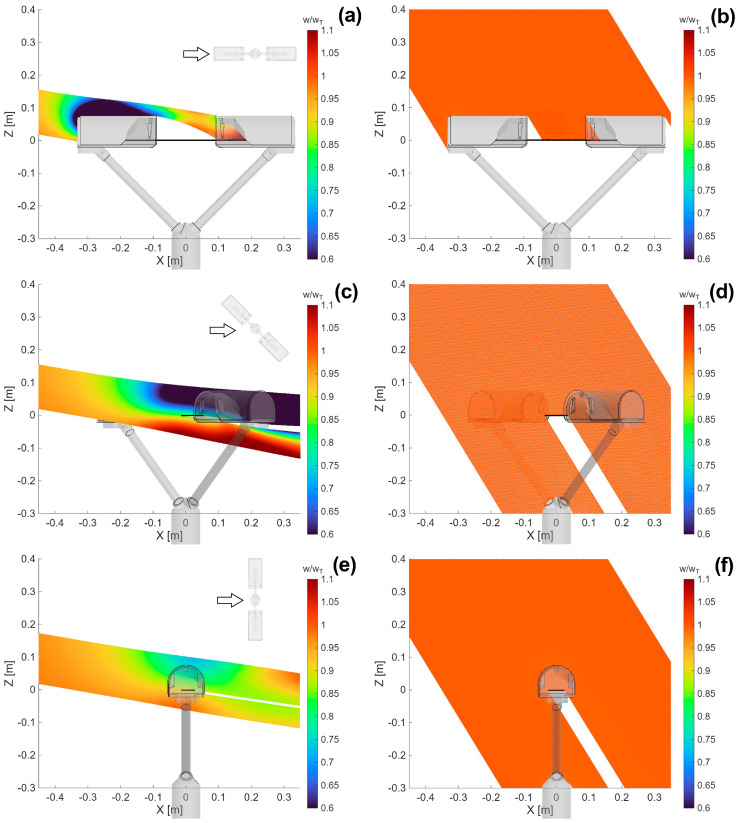
Raindrop trajectories along the Y = 0 plane, at α = 0° (**a**,**b**), 45° (**c**,**d**) and 90° (**e**,**f**) and U_ref_ = 5 m s^−1^, for two drop diameters of 0.25 mm (**a**,**c**,**e**) and 3 mm (**b**,**d**,**f**). The trajectories are colour-coded according to the normalized vertical component of the drop’s fall velocity. The black horizontal line shows the position of the instrument’s laser beam, while the black arrow shows the wind direction relative to the instrument orientation.

Figure 5 show examples from the extensive numerical dataset obtained by running the LPT model based on the airflow simulations described above. Each line of panels visualises the trajectories of drops with diameters of 0.25 mm and 3 mm at U_ref_ = 5 m s^−1^, while the wind direction varies from α = 0°, 45° and 90° in the three cases. Only trajectories falling along the Y = 0 plane are presented and are colour-coded according to the normalised vertical component of the drop’s fall velocity. Furthermore, trajectories reaching the gauge sensing area were stopped and drops exact crossing position was saved for further post processing.

Figure 5a shows the significant diversion of the trajectories of small raindrops at α = 0°. Near the stagnation point, upwind of the instrument’s head, the vertical velocity (w) of small raindrops decreases to approximately 60% of their terminal velocity (w_T_) due to the updraft generated (see Figure 3b). This effect, combined with the shielding provided by the instrument body, significantly reduces the number of drops that reach the instrument’s sensing area. Most drops cross the laser beam in positions that are shielded from vertical (undisturbed) precipitation. Large drops (Figure 5b) are less affected by wind, which causes them to follow slightly inclined trajectories. This induces limited shielding and few drops crossing the laser beam in positions that are shielded from vertical precipitation.

At an instrument orientation of 45°, no shielding occurs in the centre of its sensing area, but the vertical component of the fall velocity of small drops changes significantly (see Figure 5c). Above the instrument, trajectories are decelerated while below the instrument these are accelerated. This is due to the wake of the upstream head, which produces significant aerodynamic disturbance. A few small size raindrops cross the laser beam in areas that are shielded from vertical precipitation, while large drops are mostly unaffected (see Figure 5d).

The least diversion of raindrop trajectories occurs when the wind is perpendicular to the laser beam. Even small drops (Figure 5e) are minimally affected in this case, with their trajectories remaining largely undisturbed, particularly near the centre of the instrument’s sensing area. The maximum reduction in the vertical component of the fall velocity of small drops is approximately 25%, whereas large drops experience no significant changes in fall velocity (Figure 5f). However, in this case, no drops have trajectories that reach the laser beam in positions shielded from vertical precipitation.

## 4. The Wind-Induced Bias

### 4.1. Catch Ratio

The LPT model results can be used to obtain a non-dimensional indicator of instrument performance in windy conditions by considering the corresponding catch ratio (CR) for each raindrop size. This is defined as the ratio of the number of drops that reach the instrument’s sensing area in windy conditions to the number of drops that would reach it if the instrument were transparent to wind and raindrop trajectories, producing no aerodynamic disturbance or shielding effects. Only drops that cross the laser beam with their entire volume are considered meaning that drops partially crossing the edge of the laser beam are not counted (see, e.g., [28]).

The CR is instrument-specific and site-independent. Its variation with undisturbed wind speed, raindrop size, and dynamic characteristics is presented here by plotting the CR as a function of the undisturbed particle Reynolds number (Re_p_). Re_p_ is defined in Equation (1) of Chinchella et al. [3] as a function of the drop diameter, the particle fall velocity module, and the kinematic viscosity of air.

**Figure 6:**
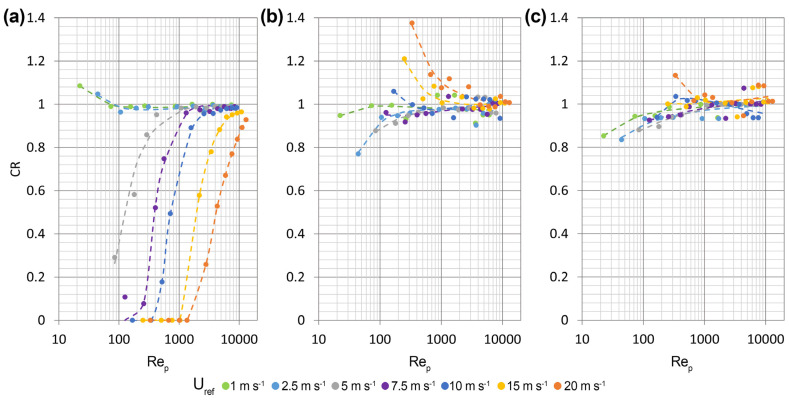
Pattern of the CR as a function of Re_p_ for different wind speed values and three wind directions: α = 0° (**a**), α = 45° (**b**) and α = 90° (**c**). The dots show the simulation results, and the dashed lines show the best-fit functions obtained as second-order inverse polynomials.

As shown in Figure 6a, the CR patterns indicate that the instrument exhibits limited overcatch of small raindrops at α = 0° for wind speeds of 1 and 2.5 m s^−1^. This overcatch disappears at values corresponding to a diameter of 0.5 mm, at which point the CR converges to unity. At stronger winds, the CR decreases rapidly as the size of the raindrops decreases and drops with a small diameter may not even reach the instrument’s sensing area (CR = 0). In these conditions, the disdrometer may completely miss the precipitation, and any drops counted are due to splashing from larger drops onto the instrument body. At a wind speed of 20 m s^−1^, even the largest drops (D = 8 mm) are slightly affected.

At α = 45° and α = 90° (Figure 6b,c), the CR values are close to one and are mostly above 0.9. A significant amount of overcatch is evident in strong winds, particularly at α = 45°. This occurs because of the significant slope of raindrop trajectories, which enables them to bypass the instrument’s shielding near its sensing area and reach the laser beam in areas that would be inaccessible to vertically falling raindrops. High variability is also evident due to the nonlinear interaction between the aerodynamic effects and shielding caused by the instrument body as a function of inclined drop trajectories. Similar results are obtained at α = 67.5°, with no overcatch observed even at high wind speeds.

For practical use, the CR values were fitted as a function of Re_p_ within the investigated range, using a second-order inverse polynomial, as follows:

(3) $$CR=a\left(U_{ref},\alpha \right)+b\left(U_{ref},\alpha \right)\cdot {{Re}_{p}}^{-1}+c\left(U_{ref},\alpha \right)\cdot {{Re}_{p}}^{0.5}$$

where the parameters *a*, *b*, and *c* are a function of the wind speed (U_ref_) and direction (α).

The best-fit values of the parameters are reported in Table A1, Table A2 and Table A3 (see Appendix A) for the combinations of wind speed and direction examined with the associated root mean square error (RMSE) listed in Table A4. The raw CR values are provided as Supplementary Materials in [29]. These curves can be used to adjust the field measurements of the OTT Parsivel^2^ disdrometer when the measured wind speed and direction are known. However, in some cases when CR = 0, measurements are lost, and adjustments must be based on further suitable assumptions (see, e.g., the work of Parasporo et al. [30]).

### 4.2. Collection and Radar Retrieval Efficiencies

Integral properties of rainfall, including rainfall intensity (RI) and radar reflectivity (Z), are commonly derived from disdrometer measurements. However, the wind-induced bias propagates into the calculation of these variables. Nevertheless, the results of the numerical simulation expressed by the CR can still be used to quantify the associated inaccuracy and provide adjustment functions.

A specific DSD formulation must be assumed so that the bias in measuring the number of drops of a given size, as provided by the CR, can be weighted according to its expected proportion in a real rainfall event. The integral must then be taken over the whole range of natural raindrop diameters. The DSD is linked to rainfall intensity since high-intensity rainfall has a higher proportion of large drops and a higher total number of drops of all sizes than low-intensity rainfall. The relationship between DSD and rainfall intensity varies depending on the location and may also change depending on the type of rainfall event (e.g., convective or stratiform).

The wind-induced bias of integral variables from a specific instrument (in this case, the OTT Parsivel^2^ disdrometer) can only be calculated using a site-dependent DSD function. This can be derived either directly from past instrument measurements (after calibration and correction of the wind-induced bias), or from literature or other instruments.

In this work, we adopted the typical exponential DSD formulation [31], using the intercept N_0_ (mm^−1^ m^−3^) and slope Λ (mm^−1^) parameters expressed as a function of RI (mm h^−1^) in the form:

(4) $$N_{0}=835.91\cdot {RI}^{0.8942}$$

(5) $$\Lambda =3.2863\cdot {RI}^{-0.076}$$

To quantify the accuracy of RI measurements, the wind-induced bias can be expressed in terms of Collection Efficiency (CE). This is defined as the ratio of the total amount of precipitation that reaches the sensing area of the instrument in windy conditions to the amount that would have reached it in the absence of any disturbance (i.e., the collected amount for unit CR).

**Figure 7:**
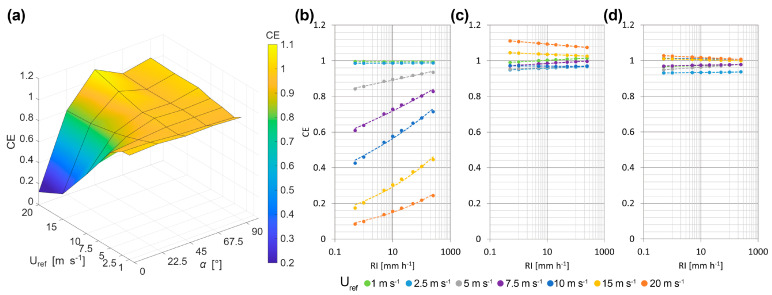
Three-dimensional CE surface for the OTT Parsivel^2^ disdrometer as a function of the undisturbed wind speed (U_ref_) and wind direction (α), assuming a sample exponential DSD [31] and computing for RI = 10 mm h^−1^ (**a**). Graphs of the CE as a function of RI for different wind speeds (colour coded) at α = 0° (**b**), α = 45° (**c**) and α = 90° (**d**).

Given the assumed DSD, the number of drops of diameter D in a cubic metre of air per unit size N(D, RI) is known, and the numerical collection efficiency can be obtained:

(6) $$CE(U_{ref},\alpha ,RI)=\frac{\int_{0}^{D_{max}}V(D)\cdot CR(D,U_{ref},\alpha )\cdot N\left(D,RI\right)dD}{\int_{0}^{D_{max}}V(D)\cdot N\left(D,RI\right)dD}$$

where V(D) is the volume of a drop with diameter D. The CE is therefore a function of the wind velocity (U_ref_), wind direction (α) and rainfall intensity (RI). Equation (6) represents the three-dimensional surface illustrated in Figure 7a for an RI of 10 mm h^−1^.

Figure 7a shows that instrument performances are mostly determined by the wind direction. The CE drops considerably as the wind speed increases for α = 0°, although some precipitation is still sensed by the instrument even at the highest wind speed. The opposite is true for the configuration at α = 45°, where the CE increases with increasing wind speed. This has already been noted in the CRs and is due to the specific shape of the instrument heads. This shape allows raindrops approaching with a shallow trajectory to cross the laser beam in positions that would normally be unreachable for vertically falling drops. For the configuration at a = 90°, the CE is almost constant and depends only slightly on the wind speed. This configuration performs best and can provide almost unbiased RI measurements.

The CE was also computed for a wide interval of RI—from 0.5 to 500 mm h^−1^—as show in Figure 7b–d. A general trend is clearly visible, where the wind-induced bias decreases with increasing RI. This can be easily explained by the fact that the number of larger drops—less affected by wind—increases with increasing the RI. Furthermore, even in the worst performing scenario (α = 0°, U_ref_ = 20 m s^−1^ and RI = 0.5 mm h^−1^) CE values are greater than 0. This—in contrast to CRs—means that, if a reliable relationship between DSD and RI is available for the measurement site, RI can always be corrected. However, this condition is still not ideal.

The power law shown in Equation (7) for the CE fits all combinations of wind speeds and directions well. The values of the two fitting parameters, *p* and *q* and the associated RMSE, are reported in Table A5, Table A6 and Table A7 (see Appendix A) for all combinations of wind speed and direction considered. By inverting Equation (7) and using the definition of CE, the rainfall intensity effectively reaching the instrument sensing area (RI_r_) can be computed from Equation (8) once the measured rainfall intensity (RI_m_) is known.

(7) $$CE=p\cdot {RI}^{q}$$

(8) $${RI}_{r}={\left(\frac{{RI}_{m}}{p}\right)}^{\frac{1}{q+1}}$$

A second integral property of precipitation is the radar reflectivity factor (dBZ). The Radar Retrieval Efficiency (RRE) is a performance parameter defined as the ratio of the radar reflectivity factor obtained from a DSD affected by wind-induced bias to the radar reflectivity factor computed using a corrected DSD. It is computed as shown in Equation (9), where $Z_{0}$ is the radar reflectivity of a single 1 mm drop in a cubic meter of air.

(9) $$RRE(U_{ref},\alpha ,RI)=\frac{\log_{10}\frac{\int_{0}^{D_{max}}D^{6}\cdot CR(D,U_{ref},\alpha )\cdot N\left(D,RI\right)dD}{Z_{0}}}{\log_{10}\frac{\int_{0}^{D_{max}}D^{6}\cdot N\left(D,RI\right)dD}{Z_{0}}}$$

**Figure 8:**
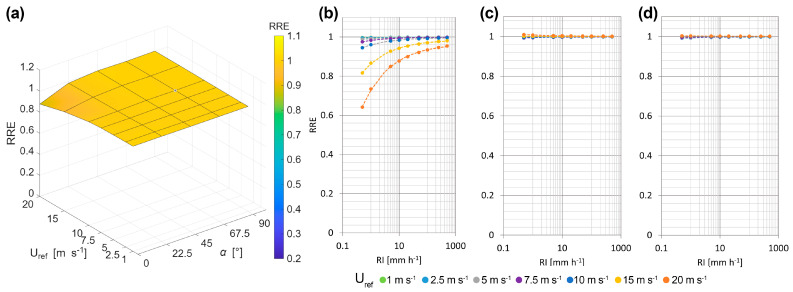
Three-dimensional RRE surface for the OTT Parsivel^2^ disdrometer as a function of the undisturbed wind speed (U_ref_) and wind direction (α), assuming a sample exponential DSD [31] and computing for RI = 10 mm h^−1^ (**a**). Graphs of the RRE as a function of RI (mm h^−1^) for different wind speeds (colour coded) at α = 0° (**b**), α = 45° (**c**) and α = 90° (**d**).

In this case, the RRE was computed by considering a reference exponential DSD (defined above) and obtaining the DSD affected by wind-induced bias by multiplying each diameter bin of the reference DSD by its corresponding CR. Figure 8a shows the RRE for RI = 10 mm h^−1^ and various combinations of wind speed and direction.

There is a limited dependency on both wind speed and direction. Lower values than unity are observed at α = 0° and high wind speed. However, for most environmental conditions, dBZ measurements are quite acceptable (especially below 10 m s^−1^ where the bias is negligible). This is because the radar reflectivity factor is mostly influenced by medium- and large-size drops, which are generally less affected by wind.

Similarly to the CE, the RRE values as a function of precipitation intensity are shown in Figure 8b–d for various wind directions and RI values ranging from 0.5 to 500 mm/h. As in the previous case, the wind-induced bias decreases as the RI increases, and the values can be fitted using the three-parameter power law shown in Equation (10).

(10) $$RRE=r+s\cdot {RI}^{t}$$

The values of the three fitting parameters, *r*, *s* and *t* and the associated RMSE, are reported in Table A8, Table A9, Table A10 and Table A11 (see Appendix A) for all considered combinations of wind speed and direction. The measured radar reflectivity factor can be corrected using RI_r_ instead of the nominal value (RI) in Equation (11).

(11) $${dBZ}_{r}=\frac{{dBZ}_{m}}{r+s\cdot {RI}^{t}}$$

## 5. Conclusions

The wind-induced bias in rainfall measurements taken with the OTT Parsivel^2^ disdrometer is significant and varies greatly depending on the wind speed and direction. Numerical simulations show that the wind significantly impacts raindrop trajectories when it is parallel to the instrument’s laser beam. Conversely, a limited—or, in some cases, negligible—impact is observed when the wind is perpendicular to the instrument. The OTT Parsivel^2^ also shows significant overestimation of the number of raindrops, which, depending on the wind direction, may decrease with increasing wind speed (α = 0°) or increase in stronger winds (α = 45°).

This behaviour is explained by the complex shape of the instrument’s emitter and receiver heads, which have large lateral protection shields. While this configuration may be effective in shielding the instrument from glare due to the sun or avoiding splashing on the optical components in the absence of wind, the shape of the heads is not optimised for windy conditions. In the case of drops falling at a strong inclination, their trajectories may hit the upstream head without even reaching the instrument’s sensing area, despite not being significantly diverted by the aerodynamic disturbance. In other cases, for α between 22.5° and 67.5°, trajectories may bypass the shield instead and cross the laser beam at positions that would not be reached by vertically falling drops. However, as the instrument’s measuring principle depends only on the blockage produced by the drop and not its longitudinal position along the laser beam, it is assumed that these drops can be sensed correctly by the instrument.

The impact of wind cannot be overlooked, and disdrometer measurements should be accompanied by additional measurements of wind speed and direction. In any case, measurements from the OTT Parsivel^2^ should be treated with caution and adjusted for wind-induced bias at any site where wind is a common occurrence during rainfall. The CR functions derived in this study can be used to quantify the measurement bias of the DSD and of any integral rainfall variable derived from it. Adjustment of integral variables can be achieved by considering the local DSD and its relationship with rainfall intensity.

Proper installation of the instrument with the laser beam perpendicular to the prevailing wind direction, as well as choosing an installation site that is protected from the wind, may help to mitigate wind-induced bias. Further mitigation of this bias would require considerable modifications to the instrument design or the use of windshields. However, adjustment of the measured data according to the correction function proposed in this work would not be possible in these latter cases, since the aerodynamic disturbance would change drastically. 

Note that the measurement accuracy investigated in the present work only refers to the wind-induced bias, while instrumental biases should be considered as well before using the derived data in any research or practical application. Further research is needed to quantify the instrumental bias of disdrometers, which would add to the accuracy assessment for this instrument. At present, only the accuracy declared by the manufacturer is available, in the absence of any agreed and/or standardised methodology for the laboratory calibration of disdrometers.

In the present work a low value of the free-stream turbulence intensity was set (equal to 1%) as representative of a well-designed measurement site. This choice allows us to focus on the effect of the aerodynamic response of the instrument, limiting the overlap of multiple effects induced by the free-stream turbulence and the turbulent structures that develop around the gauge geometry. Nevertheless, in the field the free-stream turbulence intensity can assume higher values due to the specific characteristics of the installation site. For example, direct measurements taken at a field test site in Nafferton, UK, using a 3D sonic anemometer to measure high-frequency wind, allowed the turbulence intensity at a rain gauge collector height to be quantified as between 0.1 and 0.4 at wind speeds below 6 m/s [32]. For the authors knowledge the role of free-stream turbulence was investigated in the literature for catching gauges only. Therefore, starting from the results of the present paper, the role of free-stream turbulence on disdrometer measurements will be investigated in the future.

In addition, future research will involve validating the numerical results obtained in this study by comparing the adjusted field measurements obtained using the OTT Parsivel^2^ disdrometer with the measurements obtained using a suitable reference instrument under the influence of natural wind. The choice of the reference instrument will be critical to this exercise, since it will be important to compare not only integral quantities (such as rainfall depth or intensity), but also microphysical features such as drop size and velocity distribution.

## Figures and Tables

**Table 1:** Mesh size and quality parameters for the five configurations investigated.

Wind Direction	n° Cells	Avg. Non-Orthogonality	Max Non-Orthogonality	Max Skewness	Max Aspect Ratio
0.0°	6,437,616	5.34	64.60	3.64	22.00
22.5°	7,134,222	5.39	64.95	3.99	30.99
45.0°	7,733,644	5.27	64.99	3.14	20.23
67.5°	8,157,556	5.13	64.80	4.00	30.96
90.0°	7,687,848	4.98	64.60	3.64	22.00

## Data Availability

The numerical catch ratios obtained in this work are available in [29].

## Supplementary Materials

The following supporting information can be downloaded at: https://doi.org/10.5281/zenodo.17160829, accessed on 30 September 2025, numerical catch ratios for each combination of drop diameter, wind speed, and wind direction.

## Abbreviations

The following abbreviations are used in this manuscript:

NCG	Non-Catching type Gauge
DSD	Drop Size Distribution
RI	Rainfall Intensity
KE	Kinetic Energy
CG	Catching Gauge
LPM	Laser Precipitation Monitor
2DVD	Two-Dimensional Video Disdrometer
WT	Wind Tunnel
CFD	Computational Fluid Dynamics
PWS	Present Weather Sensor
URANS	Unsteady Reynolds Averaged Navier–Stokes
SST	Shear Stress Transport
CR	Catch Ratio
CE	Collection Efficiency
RRE	Radar Retrieval Efficiency
RMSE	Root Mean Square Error

## Appendix A

**Table A1:** Numerical values of the parameter $a\left(U_{ref},\alpha \right)$ for the investigated combinations of wind speed and direction.

$a\left(U_{ref},\alpha \right)$	U_ref_ [m s^−1^]						
α	1	2.5	5	7.5	10	15	20
0.0°	0.998	0.983	0.967	0.974	0.999	0.998	0.983
22.5°	1.003	0.949	0.960	0.970	1.001	1.003	0.949
45.0°	0.983	0.946	1.002	1.011	1.027	0.983	0.946
67.5°	0.835	0.990	1.013	0.982	1.030	0.835	0.990
90.0°	0.799	1.088	1.043	0.965	0.906	0.799	1.088

**Table A2:** Numerical values of the parameter $b\left(U_{ref},\alpha \right)$ for the investigated combinations of wind speed and direction.

$b\left(U_{ref},\alpha \right)$	U_ref_ [m s^−1^]						
α	1	2.5	5	7.5	10	15	20
0.0°	4.154	−0.833	−2.892	−4.209	−1.642	4.154	−0.833
22.5°	8.064	−27.801	−14.567	−8.536	−0.758	8.064	−27.801
45.0°	−78.764	−64.881	−5.208	−10.692	2.677	−78.764	−64.881
67.5°	−473.584	−50.173	12.251	−27.927	6.658	−473.584	−50.173
90.0°	−917.038	16.279	50.275	−43.677	−66.968	−917.038	16.279

**Table A3:** Numerical values of the parameter $c\left(U_{ref},\alpha \right)$ for the investigated combinations of wind speed and direction.

$c\left(U_{ref},\alpha \right)$	U_ref_ [m s^−1^]						
α	1	2.5	5	7.5	10	15	20
0.0°	−0.469	0.254	0.518	0.384	−0.335	−0.469	0.254
22.5°	−0.953	2.457	0.967	0.206	−0.916	−0.953	2.457
45.0°	1.944	3.177	−0.629	−0.653	−1.692	1.944	3.177
67.5°	17.371	−0.521	−1.793	0.640	−1.768	17.371	−0.521
90.0°	26.074	−7.390	−3.667	1.554	5.668	26.074	−7.390

**Table A4:** RMSE resulting from the fit of the parameters $a\left(U_{ref},\alpha \right)$, $b\left(U_{ref},\alpha \right)$ and $c\left(U_{ref},\alpha \right)$.

RMSE	Uref [m s−1]						
α	1	2.5	5	7.5	10	15	20
0.0°	0.010	0.010	0.039	0.036	0.010	0.006	0.001
22.5°	0.029	0.027	0.022	0.024	0.029	0.026	0.018
45.0°	0.039	0.027	0.024	0.025	0.033	0.026	0.028
67.5°	0.022	0.017	0.018	0.017	0.011	0.015	0.014
90.0°	0.018	0.022	0.009	0.029	0.022	0.035	0.036

**Table A5:** Numerical values of the parameter $p\left(U_{ref},\alpha \right)$ for the investigated combinations of wind speed and direction.

$p\left(U_{ref},\alpha \right)$	U_ref_ [m s^−1^]						
α	1	2.5	5	7.5	10	15	20
0.0°	0.994	0.986	0.857	0.642	0.468	0.212	0.104
22.5°	1.003	0.972	0.911	0.864	0.839	0.806	0.804
45.0°	0.994	0.951	0.955	0.975	0.971	1.043	1.107
67.5°	0.982	0.941	0.953	0.956	0.949	0.949	0.962
90.0°	0.968	0.932	0.952	0.971	1.012	1.009	1.026

**Table A6:** Numerical values of the parameter $q\left(U_{ref},\alpha \right)$ for the investigated combinations of wind speed and direction.

$q\left(U_{ref},\alpha \right)$	U_ref_ [m s^−1^]						
α	1	2.5	5	7.5	10	15	20
0.0°	0.0000	0.0002	0.0169	0.0488	0.0808	0.1406	0.1605
22.5°	0.0022	0.0060	0.0069	0.0119	0.0159	0.0114	0.0041
45.0°	0.0038	0.0031	0.0031	0.0043	−0.0005	−0.0030	−0.0054
67.5°	0.0031	0.0028	0.0058	0.0052	0.0018	0.0020	0.0021
90.0°	0.0020	0.0010	0.0049	0.0014	−0.0008	−0.0018	−0.0033

**Table A7:** RMSE resulting from the fit of the parameters $p\left(U_{ref},\alpha \right)$ and $q\left(U_{ref},\alpha \right)$.

RMSE	Uref [m s−1]						
α	1	2.5	5	7.5	10	15	20
0.0°	0.00003	0.00004	0.00009	0.00017	0.00042	0.00146	0.00289
22.5°	0.00003	0.00004	0.00004	0.00015	0.00021	0.00030	0.00035
45.0°	0.00003	0.00005	0.00005	0.00006	0.00004	0.00003	0.00008
67.5°	0.00004	0.00007	0.00005	0.00005	0.00005	0.00005	0.00004
90.0°	0.00004	0.00007	0.00005	0.00004	0.00003	0.00002	0.00004

**Table A8:** Numerical values of the parameter $r\left(U_{ref},\alpha \right)$ for the investigated combinations of wind speed and direction.

$r\left(U_{ref},\alpha \right)$	U_ref_ [m s^−1^]						
α	1	2.5	5	7.5	10	15	20
0.0°	0.9994	0.9992	0.9991	0.9987	0.9966	0.9862	0.9666
22.5°	0.9994	0.9993	0.9988	0.9974	0.9975	0.9955	0.9939
45.0°	0.9986	0.9984	0.9992	0.9990	0.9997	1.0000	1.0012
67.5°	0.9987	0.9981	0.9995	0.9990	0.9986	0.9992	0.9994
90.0°	0.9994	0.9988	0.9996	0.9999	0.9991	1.0012	1.0013

**Table A9:** Numerical values of the parameter $s\left(U_{ref},\alpha \right)$ for the investigated combinations of wind speed and direction.

$s\left(U_{ref},\alpha \right)$	U_ref_ [m s^−1^]						
α	1	2.5	5	7.5	10	15	20
0.0°	−0.0021	−0.0031	−0.0080	−0.0166	−0.0372	−0.1231	−0.2380
22.5°	−0.0011	0.0001	−0.0038	−0.0121	−0.0173	−0.0243	−0.0286
45.0°	−0.0022	−0.0036	−0.0040	−0.0047	−0.0031	0.0011	0.0064
67.5°	−0.0025	−0.0053	−0.0041	−0.0039	−0.0040	−0.0038	−0.0026
90.0°	−0.0026	−0.0055	−0.0045	−0.0029	0.0022	0.0015	0.0016

**Table A10:** Numerical values of the parameter $t\left(U_{ref},\alpha \right)$ for the investigated combinations of wind speed and direction.

$t\left(U_{ref},\alpha \right)$	U_ref_ [m s^−1^]						
α	1	2.5	5	7.5	10	15	20
0.0°	−0.374	−0.377	−0.449	−0.495	−0.470	−0.452	−0.435
22.5°	−0.294	−0.135	−0.467	−0.409	−0.398	−0.386	−0.378
45.0°	−0.387	−0.408	−0.403	−0.386	−0.353	−0.437	−0.417
67.5°	−0.413	−0.400	−0.415	−0.414	−0.410	−0.397	−0.394
90.0°	−0.425	−0.401	−0.403	−0.399	−0.354	−0.363	−0.369

**Table A11:** RMSE resulting from the fit of the parameters $r\left(U_{ref},\alpha \right)$, $s\left(U_{ref},\alpha \right)$ and $t\left(U_{ref},\alpha \right)$.

RMSE	U_ref_ [m s^−1^]						
α	1	2.5	5	7.5	10	15	20
0.0°	0.0010	0.0000	0.0036	0.0085	0.0117	0.0117	0.0055
22.5°	0.0003	0.0011	0.0014	0.0018	0.0021	0.0002	0.0012
45.0°	0.0006	0.0006	0.0005	0.0011	0.0014	0.0000	0.0002
67.5°	0.0008	0.0004	0.0012	0.0011	0.0003	0.0001	0.0003
90.0°	0.0009	0.0001	0.0006	0.0006	0.0002	0.0003	0.0001
